# Predicting California bearing ratio of HARHA-treated expansive soils using Gaussian process regression

**DOI:** 10.1038/s41598-023-40903-1

**Published:** 2023-08-21

**Authors:** Mahmood Ahmad, Mohammad A. Al-Zubi, Ewa Kubińska-Jabcoń, Ali Majdi, Ramez A. Al-Mansob, Mohanad Muayad Sabri Sabri, Enas Ali, Jamil Abdulrabb Naji, Ashraf Y. Elnaggar, Bakht Zamin

**Affiliations:** 1 Department of Civil Engineering, Faculty of Engineering, International Islamic University Malaysia, Jalan Gombak, Selangor 50728 Malaysia; 2 Department of Civil Engineering, University of Engineering and Technology Peshawar (Bannu Campus), Bannu, 28100 Pakistan; 3 Department of Mechanical Engineering, Hijjawai Faculty for Engineering, Yarmouk University, Irbid, 21163 Jordan; 4 Faculty of Management, AGH University of Science and Technology, 30-067 Krakow, Poland; 5 Department of Building and Construction Techniques Engineering, Al-Mustaqbal University College, Hilla, 51001 Iraq; 6 Peter the Great St. Petersburg Polytechnic University, 195251 St. Petersburg, Russia; 7 Faculty of Engineering and Technology, Future University in Egypt, New Cairo, 11835 Egypt; 8 Department of Civil Engineering, Al-Baha University, Al-Baha, 65527 P. O. Box 1988, Saudi Arabia; 9 Department of Food Nutrition Science, College of Science, Taif University, Taif, 21944 P. O. Box 11099, Saudi Arabia; 10 Department of Civil Engineering, CECOS University of IT and Emerging Sciences, Peshawar, 25000 Pakistan

**Keywords:** Civil engineering, Engineering, Materials science

## Abstract

The California bearing ratio (CBR) is one of the basic subgrade strength characterization properties in road pavement design for evaluating the bearing capacity of pavement subgrade materials. In this research, a new model based on the Gaussian process regression (GPR) computing technique was trained and developed to predict CBR value of hydrated lime-activated rice husk ash (HARHA) treated soil. An experimental database containing 121 data points have been used. The dataset contains input parameters namely HARHA—a hybrid geometrical binder, liquid limit, plastic limit, plastic index, optimum moisture content, activity and maximum dry density while the output parameter for the model is CBR. The performance of the GPR model is assessed using statistical parameters, including the coefficient of determination (R^2^), mean absolute error (MAE), root mean square error (RMSE), Relative Root Mean Square Error (RRMSE), and performance indicator (ρ). The obtained results through GPR model yield higher accuracy as compare to recently establish artificial neural network (ANN) and gene expression programming (GEP) models in the literature. The analysis of the R^2^ together with MAE, RMSE, RRMSE, and ρ values for the CBR demonstrates that the GPR achieved a better prediction performance in training phase with (R^2^ = 0.9999, MAE = 0.0920, RMSE = 0.13907, RRMSE = 0.0078 and ρ = 0.00391) succeeded by the ANN model with (R^2^ = 0.9998, MAE = 0.0962, RMSE = 4.98, RRMSE = 0.20, and ρ = 0.100) and GEP model with (R^2^ = 0.9972, MAE = 0.5, RMSE = 4.94, RRMSE = 0.202, and ρ = 0.101). Furthermore, the sensitivity analysis result shows that HARHA was the key parameter affecting the CBR.

## Introduction

The mechanical index of geomaterials must be accurately predicted for robust pavement design^1^. The subgrade soil's strength is commonly measured by its California Bearing Ratio (CBR). CBR is a static strength and bearing capacity index that can be measured in the laboratory or in situ^2,3^. The CBR is an important input parameter for predicting the stiffness modulus of the subgrade soil, which is an essential pavement design index when cyclic loading is considered^4,5^. The CBR value is used to indirectly estimate the thickness of subgrade materials in large infrastructure projects. Consequently, precise and timely estimation of this parameter is extremely important to the design process and construction schedule.

The CBR test is a simple strength test that compares the bearing capacity of a material to that of well-graded crushed stone (a high-quality crushed stone material should have a CBR of 100%). It is intended for, but not limited to, evaluating the cohesiveness of materials with particle sizes of less than 19 mm (0.75 in). In accordance with current American Association of State Highway and Transportation Officials 2003 requirements, the laboratory CBR test entails soil mass penetration utilizing a circular 50 mm plunger applied at a rate of 1.25 mm/min^6^ into a compacted soil specimen with the optimum moisture content. The CBR test is an indirect measure of soil strength based on the resistance to penetration by a standardized piston moving at a standardized rate over a specified distance. CBR values are frequently used for highway, airport, parking lot, and other pavement designs based on empirical local or agency-specific methods. Additionally, CBR has been empirically correlated with resilient modulus and a number of other engineering soil properties.

Several studies were conducted to assess the performance of various materials, including fly ash, coarse sand, river bed material, and stone dust, that could be used to improve soft subgrades in highway construction^7–11^. For example, fly ash use in soil stabilization decreased the liquid limit and plasticity index and increased CBR^12^. Similarly, interaction between soil and waste plastic strips which causes the resistance to penetration of the plunger resulting into higher CBR values^13^.

Developing machine learning (ML) models for CBR prediction may be a viable option in this context^14^, as obtaining representative CBR values for design purposes is difficult due to insufficient soil investigations and a limited budget in determining the CBR value. In contrast, the laboratory CBR test is time-consuming and laborious. Artificial intelligence models can simulate highly nonlinear relationships between numerous input and output parameters, resulting in more precise predictions than simple and multiple regression analysis^15–17^. Several artificial intelligence model techniques have been used in engineering^18–24^ and many other disciplines^25–28^, including CBR value prediction using artificial neural network (ANN)^29^, and gene and multi expression programming^30^. As a result, this field is still being researched and investigated.

Gaussian process regression (GPR) has primarily been used in various domains of geotechnical engineering e.g.^31–41^. A critical review of the existing literature, however, indicates that, despite the successful implementation of GPR in various domains, their application to predict CBR value has not been thoroughly investigated. The purpose of this paper is to develop a new model for predicting the CBR value of expansive soil treated with hydrated lime-activated rice husk ash using the GPR computing technique. The viability and acceptability of the CBR prediction using the GPR computing method are also addressed in this paper. The dataset for this study includes seven input parameters for predicting CBR value: hydrated lime-activated rice husk ash (HARHA), liquid limit (LL), plastic limit (PL), plasticity index (PI), optimum moisture content (OMC), clay activity (CA), and maximum dry density (MDD). To compare the accuracy of the current model with that of previously developed models, several performance indexes were used, including coefficient of determination (R^2^), mean absolute error (MAE), root mean square error (RMSE), relative root mean square error (RRMSE), and performance indicator (ρ), as well as objective function (OF) to determine whether the model is overfitted or not.

The rest of the paper is structured as follows. Section “Materials and methods” presents information about the dataset, Pearson's correlation analysis, and a brief literature review on Gaussian process regression for estimating the CBR and the performance measure. Section “Results and discussion” presents the developed model's results and discussion, and Section “Limitations and future works” discusses the limitations and prospects for the future. Last Section presents the conclusions of this study.

## Materials and methods

### Dataset

**Figure 1:**
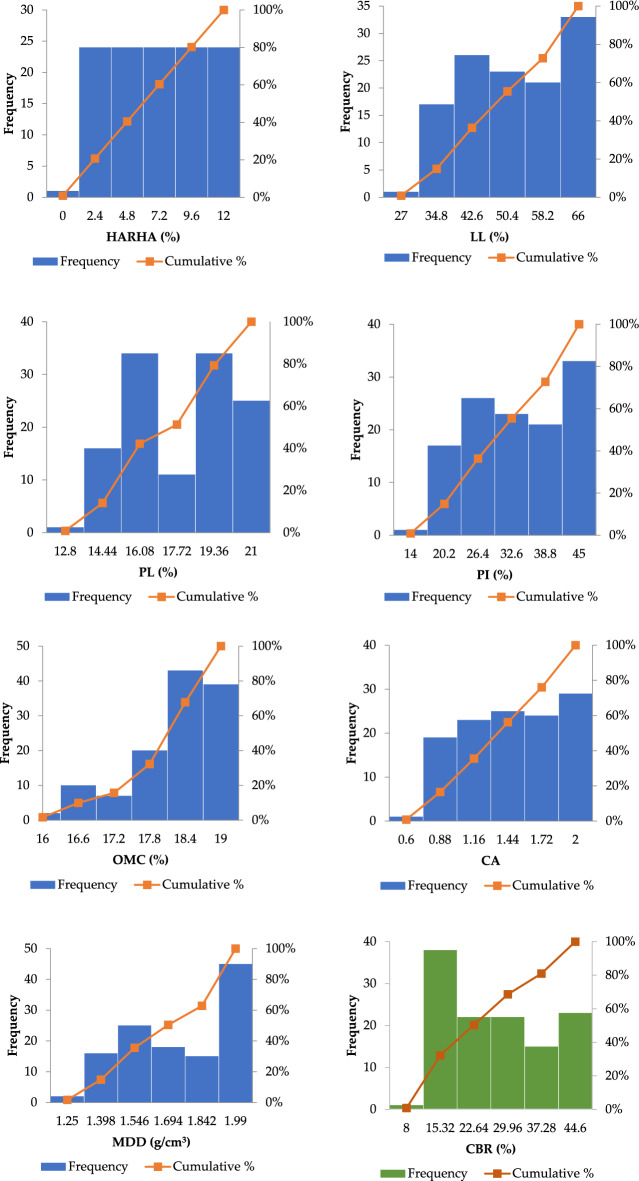
Distribution histograms for inputs (in blue) and outputs (in green).

In this study, the dataset was obtained from Onyelowe et al.^29^, which consist of 121 observations (see Appendix A, Table A1 in supplementary information file). Researchers have used a different percentage of the available data as the training set for different problems. For instance, Ahmad et al.^34^ used 70% for training and remaining 30% was equally divided into testing and validation sets. In this study, training dataset contains 85 (70%) observations while testing and validation comprises of 18 (15%) observations each. The CBR is a function of hydrated lime-activated rice husk ash (HARHA), liquid limit (LL), plastic limit (PL), plasticity index (PI), optimum moisture content (OMC), clay activity (CA), and maximum dry density (MDD)^29^. HARHA, a hybrid geometrical binder, was made by mixing rice husk with 5% hydrated lime and leaving it for 24 h to activate. Hydrated lime activates alkali, and rice husk comes from rice mills. Rice husk is agro-industrial waste. Direct combustion produces rice husk ash (RHA)^42^. Therefore, these input parameters were utilized in this study to develop the desired model. The parameters' maximum (Max), minimum (Min), mean, standard deviation (SD), and coefficient of variation (COV) were chosen in such a way that they were consistent throughout training, testing, and validation data sets (Table 1). Figure 1 illustrates the cumulative percentage and frequency distributions for all input and output parameters utilized in the CBR modeling from the aforementioned database. The cumulative percentage distribution can be used to determine what proportion of the data falls below or equals a given value. For example, if the cumulative percentage at an LL (50.4–58.2%) is 60%, then 60% of the data points are less than or equal to 20. The frequency distribution explains how data is spread across several categories or intervals. It aids in the identification of the most common or frequent values, as well as any patterns or trends. For example, if the frequency of a specific category, such as OMC (17.8–18.4%), is higher than others, it suggests that the data is concentrated in that particular region. Furthermore, readers can refer to Onyelowe et al.^29^ for additional information on carrying out the tests.

**Table 1:** Statistical parameters for data sets used for training, testing, and validation.

Parameter	Dataset	Min	Max	Mean	SD	COV
HARHA (%)	Training	0	9.2	4.2341	2.5263	6.3069
	Testing	7.6	10.2	9.1889	0.7576	0.5421
	Total	0	12	6	3.507373	58.4562
LL (%)	Training	37	66	53.9	8.0219	63.5937
	Testing	35.5	42.8	37.6444	1.9555	3.6114
	Total	27	66	47.9965	11.5363	24.0355
PL (%)	Training	14.9	21	18.3635	1.7850	3.1489
	Testing	14.9	15.9	15.1167	0.2307	0.0503
	Total	12.8	21	17.1727	2.4143	14.0587
PI (%)	Training	22	45	35.5365	6,2735	38.8943
	Testing	20.4	26.9	22.5278	1.7914	3.0309
	Total	14	45	30.8240	9.1479	29.6777
OMC (%)	Training	16	19	18.1088	0.8818	0.7683
	Testing	17.84	18.29	18.0844	0.15054	0.0214
	Total	16	19	18.024	0.7684	4.2631
CA	Training	1	2	1.5564	0.2623	0.0680
	Testing	0.86	1.19	0.9928	0.08035	0.0061
	Total	0.60	2	1.3481	0.3982	29.5361
MDD (g/cm^3^)	Training	1.25	1.964	1.5688	0.1925	0.0366
	Testing	1.85	1.982	1.9486	0.03886	0.00143
	Total	1.25	1.99	1.68	0.2432	14.4207
CBR (%)	Training	8	34.8	17.7668	7.3806	53.8322
	Testing	8.5	38.5	33.4167	6.9018	44.9892
	Total	8.00	44.6	23.8414	11.8195	49.4680

### Pearson’s correlation analysis

To determine the relationships between each pair wise variable, the Pearson correlation coefficient (*ξ*)^43^ was utilized. Table 2 detailed the relationship of all the variables based on the *ξ*. A Pearson correlation coefficient  > 0.8 indicates a strong association between each pair wise variable, values range from 0.3 to 0.8 indicate a medium relationship, and |*ξ*| < 0.30 indicates a weak relationship^44^. The rank correlation coefficient (|*ξ*|) was used to determine the associations between each pair of variables based on the distribution of the data. The parameters were determined to have a generally acceptable degree of correlation. It is evident from Table 2 that the PI is strongly correlated with CBR (|*ξ*| = 0.99514), but the OMC is weakly correlated with CBR (|*ξ*| = 0.09768) and the same is reported by Onyelowe et al.^29^. Certain variables that have a considerable amount of deviation have the potential to have an effect on prediction models^45^.

**Table 2:** Pearson’s correlation matrix.

Parameter	HARHA (%)	LL (%)	PL (%)	PI (%)	OMC (%)	CA	MDD (g/cm^3^)	CBR (%)
HARHA (%)	1.00000							
LL (%)	− 0.99724	1.00000						
PL (%)	− 0.98926	0.99152	1.00000					
PI (%)	− 0.99652	0.99941	0.98647	1.00000				
OMC (%)	0.20139	− 0.14350	− 0.17491	− 0.13480	1.00000			
CA	− 0.99388	0.99754	0.98458	0.99814	− 0.12039	1.00000		
MDD (g/cm^3^)	0.98577	− 0.98176	− 0.97696	− 0.98026	0.23936	− 0.97417	1.00000	
CBR (%)	0.99161	− 0.99425	− 0.98026	− 0.99514	0.09768	− 0.99510	0.96933	1.00000

### Gaussian processes regression (GPR)

According to Rasmussen^46^, the assumption that the GPR model operates under is that nearby observations should exchange information. Any finite number of the random variables in a Gaussian process has a joint multivariate Gaussian distribution. Let *a* × *b* stand to represent the input and output domains, respectively, from which *n* pairs (*a*_*i*_, *b*_*i*_) are randomly and uniformly distributed. For regression, let $b \subseteq \Re$; then, a Gaussian process on *a* is distinct by the mean function $\mu :a \to \Re$ and a covariance function $k:a \times a \to \Re$. The main supposition of GPR is that *y* is given as $b = f\left( a \right) + \zeta$, where $\zeta \sim N\left( {0,\sigma^{2} } \right)$. For each input *x*, there is a random variable *f*(*a*) that corresponds to the value of the stochastic function *f* at that location. In this study, it is assumed that the observational error *n* is normal, independent, and identically distributed, with a mean of zero $\mu \left( a \right) = 0$, a variance of $\sigma^{2}$, and *f*(*a*) drawn from the Gaussian process on a specified *k*. The following is,

1 $$B = \left( {b_{1} , \ldots ,b_{n} } \right)\sim N\left( {0,K + \sigma^{2} I} \right)$$

where *K*_*ij*_ = *k*(*a*_*i*_, *a*_*j*_) and* I* is the identity matrix. As $B/A\sim N\left( {0,K + \sigma^{2} I} \right)$ is normal, so is the conditional distribution of test labels given the training and test data of $p\left( {B/B,A,A*} \right)$. Then, one has $B*/B,A,A*\sim N\left( {\mu ,\sum } \right)$ where

2 $$\mu = K\left( {A*,A} \right)\left[ {K\left( {A,A} \right) + \sigma^{2} I} \right]^{ - 1} B$$

3 $$\sum = K\left( {A*,A*} \right) - \sigma^{2} I - K\left( {A*,A} \right)\left[ {K\left( {A,A} \right) + \sigma^{2} I} \right]^{ - 1} K\left( {A,A*} \right)$$

where *A* and A* represent the vectors of the training and test data respectively. The *n* × n* matrix of covariance, which is assessed at all pairs of training and test datasets, is represented by *K*(A, A*) if there are *n* training data and n* test data. Readers can get more detail information on GPR and different covariance functions from Kuss^47^.

### Evaluation measures of GPR model

To assess the GPR model's effectiveness, the evaluation measures such as coefficient of determination (R^2^), mean absolute error (MAE), root mean square error (RMSE), relative root mean square error (RRMSE), and performance indicator (ρ) are used in this study. In addition, the objective function (OF) is utilized to determine if the model has been overfitted. The mathematical expressions are given in Eqs. (4)–(9) ^30,34,48–57^.

4 $$RMSE = \sqrt {\frac{{\sum\nolimits_{i = 1}^{n} {(e_{i} - m_{i} )^{2} } }}{n}}$$

5 $$MAE = \frac{{\sum\nolimits_{i = 1}^{n} {\left| {e_{i} - m_{i} } \right|} }}{n}$$

6 $$RRMSE = \frac{1}{{\left| {\overline{e}} \right|}}\sqrt {\frac{{\sum\nolimits_{i = 1}^{n} {\left( {e_{i} - m_{i} } \right)^{2} } }}{n}}$$

7 $$R^{2} = \left[ {\frac{{\sum\nolimits_{i = 1}^{n} {\left( {e_{i} - \overline{e}_{i} } \right)\sum\nolimits_{i = 1}^{n} {\left( {m_{i} - \overline{m}_{i} } \right)} } }}{{\sqrt {\sum\nolimits_{i = 1}^{n} {\left( {e_{i} - \overline{e}_{i} } \right)^{2} \sum\nolimits_{i = 1}^{n} {\left( {m_{i} - \overline{m}_{i} } \right)^{2} } } } }}} \right]^{2}$$

8 $$\rho = \frac{RRMSE}{{1 + R}}$$

9 $$OF = \left( {\frac{{n_{T} - n_{v} }}{n}} \right)\rho_{T} + 2\left( {\frac{{n_{v} }}{n}} \right)\rho_{v}$$

where $e_{i}$ and $m_{i}$ are the *n*th measured and predicted output of the ${i}^{th}$ sample, respectively. $\overline{e}_{i}$ and $\overline{m}_{i}$ represents the average values of the measured and predicted output, respectively. The total number of datasets is shown by *n* while the training and validation datasets are shown by the subscripts *T* and *V* respectively. If a model's R^2^ values are higher than 0.8 and close to 1, it is considered as being effective^31^. The RMSE criterion measures the mean squared difference between predicted and actual output, whereas the MAE criterion measures the mean magnitude of the error. RRMSE is calculated by dividing RMSE by the measured data's mean value. To improve the performance of the model, RMSE, RRMSE and MAE should be relatively close to zero. This value cannot be 0 in practice, but the smaller it is, the more accurate the model's performance. Performance indicator (ρ) is the function of RRMSE and the coefficient of correlation (R) value^58^. The closeness of OF to zero indicates that the model is not overfit.

## Results and discussion

**Figure 2:**
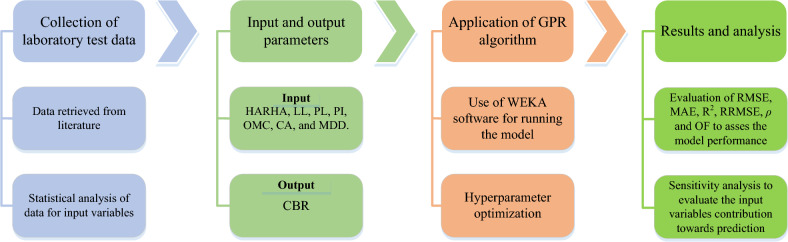
Flowchart illustrating the application of GPR to predict the CBR value.

In order to increase the accuracy and capability of the trained model Furthermore, the parameters are divided into three parts based on similar statistical characteristics, such as the mean value and coefficient of variation (COV). Model overfitting has been controlled by the mentioned validation set. The Pearson VII universal kernel known as PUK kernel function was scrutinized after multiple iteration of trial-and-error method among different function. In GPR model, the hyperparameters were fixed according to the best possible results. Hyperparameters such as noise, omega and sigma values were iterated through trial-and-error method until the desired results were achieved. Noise value was fixed at 0.3 while omega and sigma were fixed at 0.4 each listed in the following table. Figure 2 represents the flow chart of the proposed methodology in this study.

**Figure 3:**
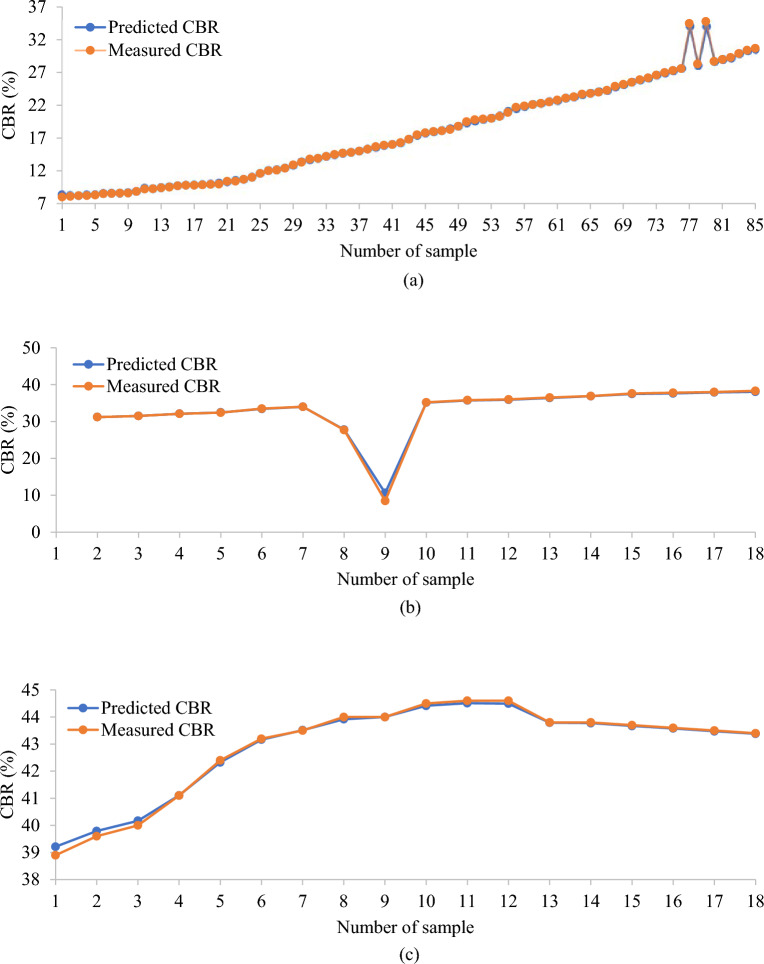
The accuracy of the GPR model in predicting CBR value in (**a**) training, (**b**) testing, and (**c**) validation sets.

To verify the effectiveness of learned models in the field of ML, models need to be assessed. Different evaluation methodologies are used with various types of models. The analysis of the built machine-learning model's predictive impact comes after the development of the machine-learning model for CBR prediction. This study verified the GPR model's CBR prediction by comparing the predicted and actual values. Figure 3 shows that there is a strong correlation between the training set's predicted value and the actual value. Although some of the data points in the test set's and validation set’s predicted value have high errors compared to the actual CBR value e.g. sample 9 (see Fig. 3b) and samples 1, 2 and 3 (see Fig. 3c) respectively, overall, the predicted value is found accurate. The findings demonstrate how well the GPR model predicts the CBR.

**Figure 4:**
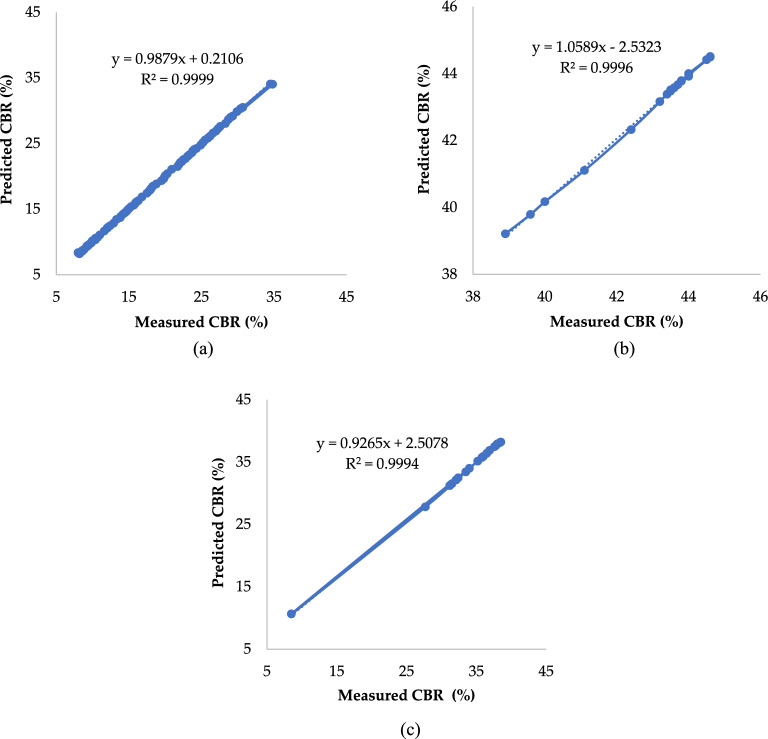
Measured and predicted CBR in the (**a**) training, (**b**) testing, and (**c**) validation sets.

Figure 4, a scatter diagram of the predicted and actual values of the training, test, and validation sets, illustrates the effect of fitting. A few points in the test set and validation set have large errors, such as in the test set, where the measure value of CBR was about 8.5% and the predicted value was as high as 10.6%; however, the small differences in individual data points have no impact on the GPR model. In addition, the CBR value is in the range of 8.2–44.5%, and predicted and actual values of the training, test, and validation sets fit well. The R^2^ value of the training set is 0.9999, the MAE value is 0.0920, the RMSE value is 0.13907, the RRMSE value is 0.0078, the ρ value is 0.00391, the R^2^ value of the test set 0.9997, the MAE value is 0.2099, the RMSE value is 0.51819, the RRMSE value is 0.0155, the ρ value is 0.00775, and the R^2^ value of the validation set 0.9996, the MAE value is 0.0719, the RMSE value is 0.1070, the RRMSE value is 0.0025, the ρ value is 0.00125. Consequently, the R^2^, MAE, RMSE, RRMSE, and ρ values of the training, test, and validation sets have common characteristics—namely, their R^2^ value is high, and their MAE, RSME, RRMSE values are low. It demonstrates that the GPR model accurately predicts the CBR value and that there is no overfitting.

The GPR model was compared to artificial neural network (ANN) and gene expression programming (GEP) models from the literature in this study. Table 3 displays the performance indexes. The summary of statistical performance in the training, testing, and validation phases shows that the MAE, RMSE, RRMSE, ρ, and OF values of the GPR model are significantly lower while the R^2^ value is larger for the CBR value. For example, in the validation stage, the analysis of the R^2^ together with MAE, RMSE, RRMSE, and ρ values for the CBR shows that the GPR model achieved better prediction results with R^2^ = 0.9996, MAE = 0.0719, RMSE = 0.1070, RRMSE = 0.0025 and ρ = 0.00125 as compared to the ANN model with R^2^ = 0.9994, MAE = 0.1649, RMSE = 1.19, RRMSE = 0.05, and ρ = 0.028) and GEP model with R^2^ = 0.9932, MAE = 0.5, RMSE = 5.49, RRMSE = 0.167 and ρ = 0.084 proposed in literature. The results indicate that the proposed model to predict CBR value using GPR was more reliable and improved for practical applications.

**Table 3:** Comparison of statistical metrics for evaluating the performance of the GPR, ANN, and GEP models.

Model	Data set	R^2^	MAE	RMSE	RRMSE	ρ	OF
GPR (this study)	Training	0.9999	0.0920	0.13907	0.0078	0.00391	0.003
	Testing	0.9997	0.2099	0.51819	0.0155	0.00775	
	Validation	0.9996	0.0719	0.1070	0.0025	0.00125	
ANN^29^	Training	0.9998	0.0962	4.98	0.20	0.100	0.077
	Testing	0.9997	0.2198	4.76	0.20	0.104	
	Validation	0.9994	0.1649	1.19	0.05	0.028	
GEP^30^	Training	0.9972	0.5	4.94	0.202	0.101	0.028
	Testing	0.9916	0.3	3.69	0.271	0.136	
	Validation	0.9932	0.5	5.49	0.167	0.084	

Sensitivity analysis is used to analyze the individual effect of input factors on CBR value. In this present study, the cosine amplitude method was used to determine the sensitivity analysis of the problem^59,60^. This method has been utilized in numerous studies^61,62^. To construct data array (*X*), data pairs are used, as follows:

10 $$X = \{ x_{1} ,x_{2} ,x_{3} , \ldots ,x_{i} , \ldots ,x_{n} \}$$

where *x*_*i*_ is a *m* length vector, a variable in the *X* array, which may be expressed as:

11 $$X = \{ x_{i1} ,x_{i2} ,x_{i3} , \ldots ,x_{im} \}$$

**Figure 5:**
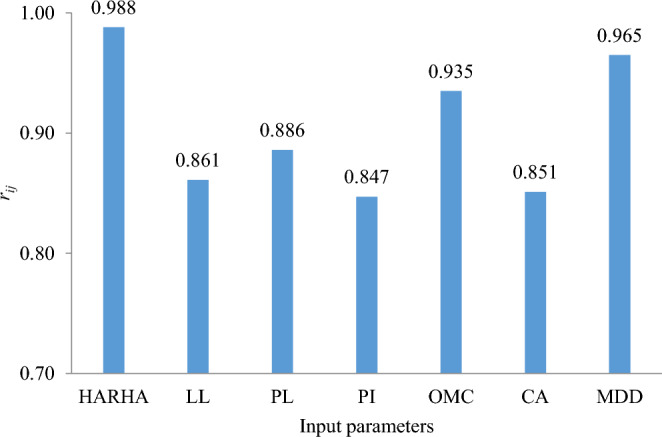
Sensitivity analysis of input variables.

The co-relation among strength of relation ${R}_{ij}$, ${x}_{i}$ and ${x}_{j}$ dataset expressed as follows:

12 $${r}_{ij}=\frac{\sum_{k=1}^{n}\left({x}_{im}\times {x}_{om}\right)}{\sqrt{\sum_{k=1}^{n}{{x}_{im}}^{2}\sum_{k=1}^{n}{{x}_{om}}^{2}}}$$

where* n* is the number of values (in this case, 85), and ${x}_{im}$ and ${x}_{om}$ are the input and output variables, respectively. The strength of the relationship (${r}_{ij}$) varies from zero to one for each input parameter. The higher the value of ${r}_{ij}$, the stronger the effect of that specific input variable on CBR value. The ${r}_{ij}$ scores for all input parameters are shown in Fig. 5. Figure 5 shows that HARHA (${r}_{ij}$ = 0.988) has the largest influence in predicting CBR value, whereas PI (${r}_{ij}$ = 0.847) has the least influence.

## Limitations and future works

It is a common fact that ML studies have always included several limitations and difficulties. One of the limitations of this study is related to the number of data samples used in the analysis, which are 121. The proposed model in this research is effective with the expected accuracy if the same input parameters are used in the future. In addition, if the same inputs are used but out of the range of our inputs, there is a possibility of an error in the analysis. In the future, more experimental data should be collected to improve the generalization capability of the proposed model. The prediction of CBR value using sophisticated ML algorithms such as deep learning is left as a topic for future study.

## Conclusions

In this research study, the GPR modeling technique was used to predict the CBR of the HARHA treated expansive soil based on the dataset characteristics. The developed GPR model's performance was evaluated using statistical metrics such as R^2^, MAE, RMSE, RRMSE, ρ, and OF, and compared to the available ANN and GEP recently developed models in the literature. The conclusions of this research can be summarized as follows:

- The new propose model of CBR using GPR achieved a better prediction performance with (R^2^ = 0.9999, MAE = 0.0920, RMSE = 0.13907, RRMSE = 0.0078, and ρ = 0.00391) succeeded by the ANN model with (R^2^ = 0.9998, MAE = 0.0962, RMSE = 4.98, RRMSE = 0.20, and ρ = 0.100) and GEP model with (R^2^ = 0.9972, MAE = 0.5, RMSE = 4.94, RRMSE = 0.202, and ρ = 0.101) in literature. The findings indicate that the GPR model predicts the CBR value of the HARHA-treated soil slightly more accurately.
- The new propose GPR model has the highest performance capability as compare to available ANN and GEP models developed recently in literature with less variation in the measured and predicted values in terms of errors in the training, test and validations sets.
- The proximal value of OF in the GPR model was 0.003 as compare to the available ANN model (0.077) and the GEP model (0.028) that were developed recently in literature which concludes that GPR model OF value ~ 0, reflects that the model is not overfitted.
- A sensitivity analysis outcome shows that HARHA was the most influential factor in predicting the CBR value.

### Supplementary Information

Supplementary Table 1.

## Data Availability

All data generated or analyzed during this study are included in its supplementary information file.

## Abbreviations

ANN: Artificial neural network
GPR: Gaussian process regression
CBR: California bearing ratio
COV: Coefficient of variation
OF: Objective function
HARHA: Hydrated lime-activated rice husk ash
LL: Liquid limit
PL: Plastic limit
PI: Plasticity index
OMC: Optimum moisture content
MDD: Maximum dry density
ML: Machine learning
Max: Maximum
Min: Minimum
CA: Clay activity
R: Correlation coefficient
SD: Standard deviation
R^2^: Coefficient of determination
MAE: Mean absolute error
RMSE: Root mean square error
RRMSE: Relative root mean square error
*ξ*: Pearson correlation coefficient
ρ: Performance indicator
$\overline{e}_{i}$: Mean of measured values
$\overline{m}_{i}$: Mean of predicted values
*n*: Total number of data
$$e_{i}$$: Measured value
$$m_{i}$$: Predicted value
$${x}_{im}$$: Input variable
$${x}_{om}$$: Output variables
*r*_*ij*_: Strength of the relation
