# Author Correction: Predicting California bearing ratio of HARHA-treated expansive soils using Gaussian process regression

**DOI:** 10.1038/s41598-023-41737-7

**Published:** 2023-09-01

**Authors:** Mahmood Ahmad, Mohammad A. Al-Zubi, Ewa Kubińska-Jabcoń, Ali Majdi, Ramez A. Al-Mansob, Mohanad Muayad Sabri Sabri, Enas Ali, Jamil Abdulrabb Naji, Ashraf Y. Elnaggar, Bakht Zamin

**Affiliations:** 1 Department of Civil Engineering, Faculty of Engineering, International Islamic University Malaysia, Jalan Gombak, Selangor 50728 Malaysia; 2 Department of Civil Engineering, University of Engineering and Technology Peshawar (Bannu Campus), Bannu, 28100 Pakistan; 3 Department of Mechanical Engineering, Hijjawai Faculty for Engineering, Yarmouk University, Irbid, 21163 Jordan; 4 Faculty of Management, AGH University of Science and Technology, 30-067 Krakow, Poland; 5 Department of Building and Construction Techniques Engineering, Al-Mustaqbal University College, Hilla, 51001 Iraq; 6 Peter the Great St. Petersburg Polytechnic University, 195251 St. Petersburg, Russia; 7 Faculty of Engineering and Technology, Future University in Egypt, New Cairo, 11835 Egypt; 8 Department of Civil Engineering, Al-Baha University, Al-Baha, 65527 P. O. Box 1988, Saudi Arabia; 9 Department of Food Nutrition Science, College of Science, Taif University, Taif, 21944 P. O. Box 11099, Saudi Arabia; 10 Department of Civil Engineering, CECOS University of IT and Emerging Sciences, Peshawar, 25000 Pakistan

Correction to: *Scientific Reports* 10.1038/s41598-023-40903-1, published online 21 August 2023

The Acknowledgements section in the original version of this Article was omitted. The Acknowledgements section now reads:

“The researchers would like to acknowledge the Deanship of Scientific Research, Taif University for funding this work.”

The original Article has been corrected.

