# Supplementary material for: Predicting California bearing ratio of HARHA-treated expansive soils using Gaussian process regression
Source: Sci Rep. 2023 Aug 21;13:13593. doi: 10.1038/s41598-023-40903-1 (PMC10442396; doi:10.1038/s41598-023-40903-1)
Supplement: Supplementary file 1 — Supplementary Table 1. [file 41598_2023_40903_MOESM1_ESM.docx]

**Appendix A**

**Table A1.** Dataset used in the present research.

| **CBR (%)** |  | **HARHA (%)** | **LL (%)** | **PL (%)** | **PI (%)** | **OMC (%)** | **CA** | **MDD (g/cm^3^)** |
| --- | --- | --- | --- | --- | --- | --- | --- | --- |
| 8 |  | 0 | 66 | 21 | 45 | 16 | 2 | 1.25 |
| 8.1 |  | 0.1 | 66 | 21 | 45 | 16 | 1.98 | 1.25 |
| 8.2 |  | 0.2 | 65.7 | 20.9 | 44.8 | 16.1 | 1.96 | 1.27 |
| 8.2 |  | 0.3 | 65.6 | 20.9 | 44.7 | 16.3 | 1.96 | 1.27 |
| 8.3 |  | 0.4 | 65.3 | 20.8 | 44.5 | 16.3 | 1.93 | 1.28 |
| 8.5 |  | 0.5 | 65 | 21 | 44 | 16.4 | 1.9 | 1.3 |
| 8.55 |  | 0.6 | 64.8 | 20.8 | 44 | 16.4 | 1.88 | 1.31 |
| 8.6 |  | 0.7 | 64.5 | 20.8 | 43.7 | 16.45 | 1.88 | 1.31 |
| 8.6 |  | 0.8 | 64.1 | 20.8 | 43.3 | 16.47 | 1.87 | 1.33 |
| 8.85 |  | 0.9 | 63.5 | 20.9 | 42.6 | 16.49 | 1.85 | 1.33 |
| 9.2 |  | 1 | 63 | 21 | 42 | 16.5 | 1.8 | 1.35 |
| 9.25 |  | 1.1 | 62.5 | 20.6 | 41.9 | 16.6 | 1.8 | 1.35 |
| 9.4 |  | 1.2 | 62.1 | 20.3 | 41.8 | 16.7 | 1.81 | 1.36 |
| 9.5 |  | 1.3 | 61.9 | 20.2 | 41.7 | 16.8 | 1.8 | 1.37 |
| 9.7 |  | 1.4 | 61.7 | 20.1 | 41.6 | 17 | 1.81 | 1.38 |
| 9.8 |  | 1.5 | 61.5 | 20 | 41.5 | 17.2 | 1.8 | 1.38 |
| 9.8 |  | 1.6 | 61.4 | 20 | 41.4 | 17.2 | 1.8 | 1.39 |
| 9.85 |  | 1.7 | 61.3 | 20 | 41.3 | 17.3 | 1.79 | 1.39 |
| 9.92 |  | 1.8 | 61.3 | 20.1 | 41.2 | 17.5 | 1.81 | 1.4 |
| 9.96 |  | 1.9 | 61.2 | 20.1 | 41.1 | 17.7 | 1.8 | 1.41 |
| 10.4 |  | 2 | 61 | 20 | 41 | 17.8 | 1.8 | 1.41 |
| 10.4 |  | 2.1 | 60.9 | 19.9 | 41 | 17.9 | 1.8 | 1.42 |
| 10.7 |  | 2.2 | 60.7 | 19.7 | 41 | 17.9 | 1.8 | 1.42 |
| 11 |  | 2.3 | 60.6 | 19.6 | 41 | 18 | 1.8 | 1.425 |
| 11.6 |  | 2.4 | 60.4 | 19.4 | 41 | 18.2 | 1.8 | 1.43 |
| 12 |  | 2.5 | 60 | 19 | 41 | 18.3 | 1.8 | 1.43 |
| 12.1 |  | 2.6 | 59.8 | 19 | 40.8 | 18.35 | 1.79 | 1.435 |
| 12.4 |  | 2.7 | 59.7 | 19.1 | 40.6 | 18.4 | 1.77 | 1.45 |
| 12.9 |  | 2.8 | 59.5 | 19.1 | 40.4 | 18.45 | 1.75 | 1.455 |
| 13.3 |  | 2.9 | 59.2 | 19 | 40.2 | 18.5 | 1.72 | 1.46 |
| 13.8 |  | 3 | 59 | 19 | 40 | 18.5 | 1.7 | 1.46 |
| 13.9 |  | 3.1 | 58.8 | 19.2 | 39.6 | 18.55 | 1.7 | 1.47 |
| 14.2 |  | 3.2 | 58.4 | 18.9 | 39.5 | 18.6 | 1.7 | 1.475 |
| 14.5 |  | 3.3 | 57.9 | 19.1 | 38.8 | 18.7 | 1.71 | 1.48 |
| 14.7 |  | 3.4 | 57.4 | 19 | 38.4 | 18.75 | 1.69 | 1.484 |
| 14.8 |  | 3.5 | 57 | 19 | 38 | 18.8 | 1.7 | 1.49 |
| 15 |  | 3.6 | 56.8 | 18.9 | 37.9 | 18.85 | 1.69 | 1.5 |
| 15.3 |  | 3.7 | 56.7 | 19 | 37.7 | 18.9 | 1.65 | 1.51 |
| 15.7 |  | 3.8 | 56.5 | 18.9 | 37.6 | 18.93 | 1.64 | 1.51 |
| 15.9 |  | 3.9 | 56.3 | 19 | 37.3 | 18.98 | 1.61 | 1.52 |
| 16 |  | 4 | 56 | 19 | 37 | 19 | 1.6 | 1.52 |
| 16.3 |  | 4.1 | 55.7 | 19 | 36.7 | 19 | 1.59 | 1.53 |
| 16.8 |  | 4.2 | 54.9 | 18.7 | 36.2 | 19 | 1.57 | 1.54 |
| 17.5 |  | 4.3 | 54.1 | 18.5 | 35.6 | 19 | 1.55 | 1.55 |
| 17.8 |  | 4.4 | 53.6 | 18.4 | 35.2 | 19 | 1.52 | 1.56 |
| 18 |  | 4.5 | 53 | 18 | 35 | 19 | 1.5 | 1.57 |
| 18.1 |  | 4.6 | 52.8 | 18 | 34.8 | 18.98 | 1.5 | 1.58 |
| 18.3 |  | 4.7 | 52.7 | 18 | 34.7 | 18.96 | 1.5 | 1.59 |
| 18.8 |  | 4.8 | 52.6 | 18.1 | 34.5 | 18.93 | 1.5 | 1.6 |
| 19.5 |  | 4.9 | 52.3 | 18 | 34.3 | 18.91 | 1.5 | 1.61 |
| 19.8 |  | 5 | 52 | 18 | 34 | 18.9 | 1.5 | 1.61 |
| 19.9 |  | 5.1 | 51.5 | 17.7 | 33.8 | 18.88 | 1.48 | 1.62 |
| 20 |  | 5.2 | 51.1 | 17.7 | 33.4 | 18.86 | 1.46 | 1.63 |
| 20.3 |  | 5.3 | 50.8 | 18.1 | 32.7 | 18.84 | 1.43 | 1.64 |
| 20.9 |  | 5.4 | 50.3 | 18 | 32.3 | 18.82 | 1.41 | 1.65 |
| 21.7 |  | 5.5 | 50 | 18 | 32 | 18.8 | 1.4 | 1.65 |
| 21.9 |  | 5.6 | 49.9 | 18 | 31.9 | 18.78 | 1.4 | 1.66 |
| 22.1 |  | 5.7 | 49.6 | 17.9 | 31.7 | 18.75 | 1.41 | 1.67 |
| 22.3 |  | 5.8 | 49.4 | 17.9 | 31.5 | 18.71 | 1.42 | 1.67 |
| 22.5 |  | 5.9 | 49.1 | 17.7 | 31.4 | 18.65 | 1.41 | 1.68 |
| 22.8 |  | 6 | 49 | 18 | 31 | 18.6 | 1.4 | 1.69 |
| 23.1 |  | 6.1 | 48.6 | 17.8 | 30.8 | 18.55 | 1.38 | 1.7 |
| 23.3 |  | 6.2 | 48.3 | 17.6 | 30.7 | 18.48 | 1.37 | 1.71 |
| 23.7 |  | 6.3 | 47.7 | 17.3 | 30.4 | 18.6 | 1.35 | 1.72 |
| 23.8 |  | 6.4 | 47.2 | 17 | 30.2 | 18.44 | 1.33 | 1.73 |
| 24 |  | 6.5 | 47 | 17 | 30 | 18.4 | 1.3 | 1.74 |
| 24.3 |  | 6.6 | 46.8 | 17.1 | 29.7 | 18.4 | 1.31 | 1.75 |
| 24.9 |  | 6.7 | 46.5 | 16.8 | 29.7 | 18.41 | 1.31 | 1.76 |
| 25.2 |  | 6.8 | 45.6 | 15.9 | 29.7 | 18.4 | 1.3 | 1.77 |
| 25.5 |  | 6.9 | 45.2 | 15.9 | 29.3 | 18.41 | 1.3 | 1.78 |
| 25.9 |  | 7 | 45 | 16 | 29 | 18.4 | 1.3 | 1.78 |
| 26.2 |  | 7.1 | 44.8 | 16.3 | 28.5 | 18.39 | 1.29 | 1.79 |
| 26.6 |  | 7.2 | 44.3 | 16.1 | 28.2 | 18.37 | 1.27 | 1.8 |
| 27 |  | 7.3 | 43.7 | 15.9 | 27.8 | 18.35 | 1.26 | 1.81 |
| 27.3 |  | 7.4 | 43.4 | 16 | 27.4 | 18.32 | 1.23 | 1.83 |
| 27.6 |  | 7.5 | 43 | 16 | 27 | 18.3 | 1.2 | 1.84 |
| 27.7 |  | 7.6 | 42.8 | 15.9 | 26.9 | 18.29 | 1.19 | 1.85 |
| 28.3 |  | 7.7 | 42.4 | 16 | 26.4 | 18.28 | 1.18 | 1.86 |
| 28.5 |  | 7.8 | 41.8 | 15.4 | 26.4 | 18.26 | 1.16 | 1.87 |
| 28.7 |  | 7.9 | 41.5 | 15.4 | 26.1 | 18.23 | 1.14 | 1.87 |
| 29 |  | 8 | 41 | 15 | 26 | 18.2 | 1.13 | 1.88 |
| 29.3 |  | 8.1 | 40.7 | 14.9 | 25.8 | 18.2 | 1.12 | 1.88 |
| 29.9 |  | 8.2 | 40.3 | 15 | 25.3 | 18.2 | 1.11 | 1.89 |
| 30.4 |  | 8.3 | 39.8 | 15.1 | 24.7 | 18.2 | 1.11 | 1.9 |
| 30.7 |  | 8.4 | 39.3 | 15 | 24.3 | 18.21 | 1.1 | 1.9 |
| 31.2 |  | 8.5 | 39 | 15 | 24 | 18.2 | 1 | 1.91 |
| 31.5 |  | 8.6 | 38.8 | 15 | 23.8 | 18.2 | 1 | 1.92 |
| 32.1 |  | 8.7 | 38.3 | 14.9 | 23.4 | 18.2 | 1 | 1.93 |
| 32.4 |  | 8.8 | 37.9 | 15.2 | 22.7 | 18.2 | 1 | 1.94 |
| 33.5 |  | 8.9 | 37.5 | 15.2 | 22.3 | 18.2 | 1 | 1.95 |
| 34 |  | 9 | 37 | 15 | 22 | 18.2 | 1 | 1.96 |
| 34.5 |  | 9.1 | 37 | 15 | 22 | 18.19 | 1 | 1.962 |
| 34.8 |  | 9.2 | 37 | 15 | 22 | 18.18 | 1 | 1.964 |
| 35.2 |  | 9.3 | 37 | 15 | 22 | 18.16 | 1 | 1.966 |
| 35.8 |  | 9.4 | 37 | 15 | 22 | 18.13 | 1 | 1.969 |
| 36 |  | 9.5 | 37 | 15 | 22 | 18.1 | 1 | 1.97 |
| 36.5 |  | 9.6 | 36.8 | 15.1 | 21.7 | 18 | 0.99 | 1.972 |
| 36.9 |  | 9.7 | 36.7 | 15.1 | 21.6 | 17.92 | 0.98 | 1.973 |
| 37.6 |  | 9.8 | 36.5 | 15.1 | 21.4 | 17.93 | 0.97 | 1.975 |
| 37.8 |  | 9.9 | 36.3 | 15.2 | 21.1 | 17.91 | 0.94 | 1.977 |
| 38 |  | 10 | 36 | 15 | 21 | 17.9 | 0.9 | 1.98 |
| 38.3 |  | 10.1 | 35.7 | 14.9 | 20.8 | 17.88 | 0.88 | 1.98 |
| 38.5 |  | 10.2 | 35.5 | 15.1 | 20.4 | 17.84 | 0.86 | 1.982 |
| 38.9 |  | 10.3 | 34.6 | 14.9 | 19.7 | 17.79 | 0.84 | 1.984 |
| 39.6 |  | 10.4 | 33.3 | 14 | 19.3 | 17.73 | 0.82 | 1.987 |
| 40 |  | 10.5 | 33 | 14 | 19 | 17.7 | 0.8 | 1.99 |
| 41.1 |  | 10.6 | 32.8 | 14 | 18.8 | 17.7 | 0.79 | 1.99 |
| 42.4 |  | 10.7 | 32.4 | 13.9 | 18.5 | 17.71 | 0.78 | 1.99 |
| 43.2 |  | 10.8 | 31.5 | 13.9 | 17.6 | 17.71 | 0.75 | 1.99 |
| 43.5 |  | 10.9 | 31.1 | 14 | 17.1 | 17.7 | 0.72 | 1.99 |
| 44 |  | 11 | 31 | 14 | 17 | 17.7 | 0.7 | 1.99 |
| 44 |  | 11.1 | 30.7 | 13.9 | 16.8 | 17.68 | 0.7 | 1.99 |
| 44.5 |  | 11.2 | 30.3 | 13.7 | 16.6 | 17.63 | 0.71 | 1.99 |
| 44.6 |  | 11.3 | 29.8 | 13.4 | 16.4 | 17.57 | 0.71 | 1.99 |
| 44.6 |  | 11.4 | 29.4 | 13.2 | 16.2 | 17.53 | 0.71 | 1.98 |
| 43.8 |  | 11.5 | 29 | 13 | 16 | 17.5 | 0.7 | 1.97 |
| 43.8 |  | 11.6 | 28.7 | 12.8 | 15.9 | 17.5 | 0.69 | 1.97 |
| 43.7 |  | 11.7 | 28.5 | 13 | 15.5 | 17.4 | 0.67 | 1.96 |
| 43.6 |  | 11.8 | 27.8 | 13 | 14.8 | 17.3 | 0.65 | 1.96 |
| 43.5 |  | 11.9 | 27.6 | 13.2 | 14.4 | 17.2 | 0.62 | 1.95 |
| 43.4 |  | 12 | 27 | 13 | 14 | 17.1 | 0.6 | 1.95 |
